# Risk Factors and Pregnancy Outcomes of Antepartum Hemorrhage in Women with Placenta Previa

**DOI:** 10.1007/s43032-023-01191-2

**Published:** 2023-03-20

**Authors:** Do Hwa Im, Young Nam Kim, Eun Hye Cho, Da Hyun Kim, Jung Mi Byun, Dae Hoon Jeong

**Affiliations:** 1 Department of Obstetrics and Gynecology, Inje University Busan Paik Hospital, 75 Bokji-Ro, Busanjin-Gu, Busan, 473920 South Korea; 2 Paik Institute for Clinical Research, Busan Paik Hospital, Inje University, Busan, South Korea

**Keywords:** Antepartum bleeding, Cesarean section, Neonatology, Placental pathology, Preterm labor

## Abstract

Placenta previa (PP) is one such complication related to several adverse pregnancy outcomes. Adverse outcomes are likely greater if PP coexists with antepartum hemorrhage (APH). This study aims to evaluate the risk factors and pregnancy outcomes of APH in women with PP. This retrospective case–control study included 125 singleton pregnancies with PP who delivered between 2017 and 2019. Women with PP were divided into two groups: PP without APH (*n* = 59) and PP with APH (*n* = 66). We investigated the risk factors associated with APH and compared the differences between both groups in placental histopathology lesions due to APH and the resulting maternal and neonatal outcomes. Women with APH had more frequent antepartum uterine contractions (33.3% vs. 10.2%, *P* = .002) and short cervical length (< 2.5 cm) at admission (53.0% vs. 27.1%, *P* = .003). The placentas from the APH group had lower weight (442.9 ± 110.1 vs. 488.3 ± 117.7 g, *P* = .03) in the gross findings, and a higher rate of villous agglutination lesions (42.4% vs. 22.0%, *P* = .01) in the histopathologic findings. Women with APH in PP had higher rates of composite adverse pregnancy outcomes (83.3% vs. 49.2%, *P* = .0001). Neonates born to women with APH in PP had worse neonatal outcomes (59.1% vs. 23.9%, *P* = .0001). Preterm uterine contractions and short cervical length were the most significant risk factors for APH in PP.

## Introduction

Placenta previa (PP) is defined as implantation of the placenta in the lower uterine segment wherein the placenta covers the internal cervical os or is situated very close to it [1]. PP occurs in approximately 0.3–0.5% of all pregnancies; the incidence rate has increased in recent years due to increase previous caesarean deliveries or other uterine surgeries, such as myomectomy or curettage, advanced maternal age, multiparity, and smoking [2, 3]. Pregnancy complications such as PP are related to poor maternal and neonatal morbidities including preterm deliveries, low Apgar scores, maternal hemorrhage, the need for blood transfusion, and hysterectomy [1, 4–6]. PP is also strongly associated with antepartum hemorrhage (APH). Studies have reported that APH has a prevalence of 50–52% in women with PP [7, 8]. The major complications associated with APH in women with PP include preterm delivery, blood transfusion, intensive care unit admissions, hysterectomy, and maternal death [1, 2, 9]. The mechanism of bleeding associated with PP has not yet been clearly identified, but it has been reported that abnormal placentation in the lower uterine segment can be easily separated from the decidua basalis due to uterine contraction; abnormally formed blood vessels in the decidua tissue can cause massive bleeding owing to uterine contraction [10, 11]. There is a growing interest in studying the risk factors for PP and APH, but no systematic studies have been conducted yet. This study aimed to investigate the risk factors associated with APH by analyzing the clinical characteristics and ultrasound findings of patients with APH. We also aimed to compare the differences in placental histopathological lesions due to APH and the resulting maternal and neonatal outcomes between patients with and without APH.

## Methods

### Study Population

This retrospective case–control study included 125 singleton pregnancies with PP that delivered between 24 and 42 weeks of gestation at Busan Paik Hospital between January 2017 and December 2019. The institutional review board of Inje University Busan Paik Hospital approved this study and all protocols (approval number: 2021–02-036). Participants were divided into PP with APH (*n* = 66) and PP without APH (*n* = 59). Patients with multiple pregnancies, no antenatal care, gestational age < 24 weeks, and no available medical records were excluded.

We evaluated maternal clinical characteristics, such as age, gestational age at delivery, parity, obesity, gestational diabetes mellitus, pregestational diabetes mellitus, preeclampsia, smoking status, myoma, adenomyosis, previous cesarean delivery, in vitro fertilization, prior dilatation and curettage, and antepartum uterine contractions. Pre-pregnancy obesity was defined as a pre-pregnancy body mass index (BMI) > 25 kg/m^2^, according to the World Health Organization definition for Asian populations. Antepartum uterine contractions were defined as three or more contractions per 30 min occurring at the gestational age between 24 and 34 weeks.

### Ultrasound Examination

Ultrasound assessment was performed using transvaginal and transabdominal ultrasonography in all the cases. All examinations were performed using a 4.0–6.0-MHz curved transabdominal or 6.0–12.0-MHz transvaginal transducer Voluson E8 system (GE health care, Zipf, Austria), also equipped for pulsed Doppler and color Doppler imaging.

Using the new classification, PP is diagnosed when the placenta covers or just reaches the internal cervical os. If the inferior placental edge encroaches upon the cervix, such that it is within 2 cm of the internal os but does not overlie it, the diagnosis is low-lying placenta [12–14]. The placenta was examined for location, distance from the internal os to the edge of the placenta, and the presence of adherent placental lesions. Diagnosis of adherent placenta was performed using ultrasound markers for placenta accreta spectrum (PAS) disorders, and the sonographic findings are as follows: loss of the retroplacental clear space, four or more lacunar spaces, myometrial thinning (thickness less than 1 mm), irregular bladder wall with increased vascularity, and presence of the cervix jellyfish sign [15]. Cervical examinations were performed using a standardized technique. A sagittal plane was obtained to visualize the full- length cervix. The cervical length was measured three times, and the shortest value was recorded.

### Placental Pathology

We compared the gross and microscopic findings of the placentas between the APH and non-APH groups. All observations were performed by two pathologists. The gross findings included placental weight, fetoplacental weight, diameter, thickness, chorionic plate area, placental infarction, and umbilical cord insertion site. Six sections from the placenta were divided into five slides: disc (three slides), membrane (one slide), and umbilical cord (two sections in one slide). Pathological findings were reported according to the classification criteria adopted by the Society for Pediatric Pathology (SPP) (2005) [16]. The histological data of the placenta were divided into four major categories: amniotic fluid infection, maternal malperfusion, fetal vascular thrombo-occlusive disease, and chronic inflammatory lesions. Amniotic fluid infections included chorioamniotic maternal responses (acute subchorionitis/chorionitis and acute, necrotizing, and subacute chorioamnionitis) and fetal inflammatory responses (umbilical phlebitis/chorionic vasculitis, umbilical arteritis, necrotizing funisitis). Maternal malperfusion included villous changes (placental infarction, increased syncytial knots, villous agglutination, increased intervillous fibrin, decreased placental weight/increased fetoplacental weight ratio, and distal villous hypoplasia) and vascular changes (persistent muscularization of the basal plate arteries, mural hypertrophy of the decidual arterioles, acute atherosis of the basal plate arteries, and decidual vessel thrombus). Fetal vascular thrombo-occlusion included villous changes (villous stromal-vascular karyorrhexis, hyalinized avascular villi, and fetal thrombotic vasculopathy) and vascular lesions (thrombi and/or intimal fibrin cushions of large fetal vessels, and fibromuscular sclerosis of intermediate-sized fetal vessels). Chronic inflammatory lesions included chronic villitis, chronic deciduitis, or chronic chorioamnionitis.

### Perinatal Outcomes

We evaluated the maternal and neonatal outcomes in the APH and non-APH groups. Maternal pregnancy outcome variables included maternal death, antenatal corticosteroid use, antenatal tocolytic use, emergency cesarean section, placental abruption, postpartum hemorrhage, intrauterine tamponade, cesarean hysterectomy, postpartum anemia (hemoglobin < 8), massive transfusion, and postoperative intensive care unit (ICU) admission. Composite adverse pregnancy outcome was defined as one or more of the above complications.

Neonatal complication variables included neonatal death, preterm birth, neonatal intensive care unit (NICU) admission, Apgar score at 5 min of birth, mechanical ventilation, respiratory distress syndrome (RDS), intraventricular hemorrhage (IVH), necrotizing enterocolitis (NEC), sepsis, and seizure. Neonatal death was defined as the death of a live-born neonate within 28 days of delivery. Composite adverse neonatal outcome included one or more of the above complications.

### Statistics

Data were analyzed using the MedCalc version 11.0 software (Frank Schoonjans, University of Gent, Belgium). Continuous variables were calculated using the Student’s *t*-test. Categorical variables were compared using the chi-squared test. Multivariable logistic regression was performed to investigate independent risk factors for APH. The results were considered statistically significant when the *P*-value was < 0.05.

## Results

Maternal characteristics of the study population are presented in Table 1. Their characteristics were analyzed by dividing them into baseline and antepartum parts. There were no significant differences in maternal age, parity, obesity, or history of cesarean delivery between the two groups. Compared to the PP without APH group, the PP with APH group had higher rates of in vitro fertilization, and smoking (16.7% vs. 5.1%, *P* ≤ 0.04 and 3.4% vs. 0%, *P* = 0.04, respectively). In antepartum characteristics, the PP with APH group had more antepartum uterine contractions, corticosteroid use, and tocolytic use (33.3% vs. 10.2%, *P* = 0.002; 21.2% vs. 6.8%, *P* = 0.022; and 37.9% vs. 11.9%, *P* = 0.001, respectively).

**Table 1:** Maternal characteristics placenta previa with APH and without APH

	Placenta previa with APH (*N* = 66) *n*/*N* (%)	Placenta previa without APH (*N* = 59) *n*/*N* (%)	*P* value
Baseline characteristics			
Maternal age (years)	35.1 ± 4.1	34.5 ± 3.4	.38
Elderly^†^	39 (59.1)	28 (47.5)	.19
Nulliparity	24 (36.4)	27 (45.8)	.28
Obesity^‡^	10 (15.2)	9 (15.3)	.98
In vitro fertilization	11 (16.7)	3 (5.1)	.04
Previous cesarean delivery	18 (27.3)	17 (28.8)	.84
1	13 (19.7)	13 (22.0)	.74
≥ 2	5 (7.6)	4 (6.8)	.86
Prior dilatation and curettage	31 (47.0)	20 (33.9)	.13
Uterine myoma	6 (9.1)	1 (1.7)	.07
Adenomyosis	3 (4.5)	2 (3.4)	.74
Smoking	4 (3.4)	0	.044
Antepartum characteristics			
Antepartum uterine contraction	22 (33.3)	6 (10.2)	.002
Antepartum corticosteroid use	14 (21.2)	4 (6.8)	.02
Antepartum tocolytics use	25 (37.9)	7 (11.9)	.001
Gestational diabetes mellitus	6 (9.1)	7 (11.9)	.61
Pregestational diabetes mellitus	0	0	.53
Preeclampsia	2 (3.0)	1 (1.7)	.62

Continuous variables are presented as mean ± SD. Categorical variables are presented as number (proportions)

^†^Elderly: maternal age ≥ 35 years old

^‡^Obesity: pre-pregnancy BMI (kg/m^2^) ≥ 25

The ultrasonographic findings (Table 2) showed that short cervical length at admission (< 2.5 cm) was highly associated with APH (53.0% vs. 27.1%, *P* = 0.003), but there were no significant differences between both groups regarding location of the placentas, type of PP, and findings of adherent placenta.

**Table 2:** Ultrasonographic findings

	Placenta previa with APH (*N* = 66) *n*/*N* (%)	Placenta previa without APH (*N* = 59) *n*/*N* (%)	*P* value
Classification			
Placenta previa	19 (28.8)	16 (27.1)	.83
Low-lying placenta	47 (71.2)	43 (72.9)	
Placentation site			
Anterior wall	55 (83.3)	43 (72.9)	.15
Lateral or posterior wall	11 (16.7)	16 (27.1)	
Adherent placenta^†^	32 (48.5)	20 (33.9)	.09
Short cervical length at admission (< 2.5 cm)	35 (53.0)	16 (27.1)	.003

Continuous variables are presented as mean ± SD. Categorical variables are presented as number (proportions)

^†^Adherent placenta included any of the following: loss of the retroplacental clear space, 4 or more lacunar spaces, myometrial thinning (thickness less than 1 mm), irregular bladder wall with increased vascularity, and cervix jellyfish sign

We analyzed various factors to identify the risk factors associated with PP with APH using multivariable logistic regression analysis. Antepartum uterine contraction [odds ratio (OR) = 4.15, 95% confidence interval (CI) = 1.51–11.49, *P* = 0.01] and short cervical length at admission (< 2.5 cm) (OR = 2.68, 95% CI = 1.13–6.30, *P* = 0.02) were found to be the most significant risk factors for APH (Table 3).

**Table 3:** Risk factors for antepartum hemorrhage—multivariable logistic analysis

	OR (95% CI)	*P* value
Antepartum uterine contraction	4.15 (1.51–11.49)	.01
Short cervical length at admission (< 2.5 cm)	2.68 (1.13–6.30)	.02
In vitro fertilization	3.46 (0.87–13.83)	.07
Previous cesarean delivery	0.85 (0.38–2.09)	.72
Complete placenta previa	1.73 (0.76–3.95)	.18
Adherent placenta	1.41 (0.64–3.26)	.41
Elderly	1.42 (0.65–3.09)	.55

Table 4 shows the placental gross and histologic findings between the two groups. In the gross findings of the placenta, the PP with APH group had lower placental weight (442.9 ± 110.1 vs. 488.3 ± 117.7 gm, *P* = 0.03), smaller mean chorionic diameter (17.5 ± 2.9 vs. 18.9 ± 3.1 cm, *P* = 0.01), and reduced chorionic plate area (199.9 ± 58.4 vs. 236.7 ± 75.6 cm, *P* = 0.003). There were also significant differences between the two groups when the analysis was performed after adjusting for gestational age at delivery. No significant differences were observed between the groups regarding marginal cord insertion and placental infarction. In the histopathological findings, placental lesions consistent with maternal malperfusion lesions, especially villous agglutination, were more frequent in the PP with APH group than in the PP without APH group (42.4% vs. 22.0%, *P* = 0.01). The rate of amniotic infection lesions, fetal vascular thrombo-occlusive lesions, and chronic inflammatory lesions did not differ between the two groups.

**Table 4:** Placental gross and histopathologic findings

	Placenta previa with APH (*N* = 66) *n*/*N* (%)	Placenta previa without APH (*N* = 59) *n*/*N* (%)	*P* value	*aP* value
Gross findings				
Placental weight (grams)	442.9 $\pm 110.1$	488.3 $\pm 117.7$	.03	.003
Fetoplacental weight (ratio)	6.1 $\pm$ 1.7	$6.4\pm 1.5$	.33	.0001
Largest diameter of chorionic plate (cm)	17.5 $\pm$ 2.9	18.9 $\pm$ 3.1	.01	< .0001
Smallest diameter of chorionic plate (cm)	14.2 $\pm$ 2.7	15.6 $\pm 3.3$	.01	.01
Placenta thickness (cm^2^)	2.8 $\pm 1.2$	2.6 $\pm$ 1.1	.30	.05
Chorionic plate area (cm^2^)	199.9 $\pm$ 58.4	236.7 $\pm$ 75.6	.003	.0001
Marginal cord insertion (%)	8 (12.1)	4 (6.8)	.31	.20
Placental infarction (%)	20 (30.3)	20 (33.9)	.67	.16
Histopathologic findings				
Findings consistent with amniotic fluid infection	10 (15.2)	4 (6.8)	.14	
Chorioamnionitis maternal response	8 (12.1)	4 (6.8)	.31	
Chorioamnionitis fetal response	3 (4.5)	1 (1.7)	.36	
Findings consistent with maternal malperfusion	53 (80.3)	38 (64.4)	.04	
Villous infarct	27 (40.9)	27 (45.8)	.58	
Increased syncytial knots	6 (9.1)	3 (5.1)	.38	
Villous agglutination	28 (42.4)	13 (22.0)	.01	
Increased intervillous fibrin	11 (16.7)	5 (8.5)	.17	
Distal villous hypoplasia	5 (7.6)	1 (1.7)	.12	
Vascular change	1 (1.5)	1 (1.7)	.93	
Findings consistent with fetal vascular thrombo-occlusion disease	2 (3.0)	7 (11.9)	.05	
Villous change	2 (3.0)	5 (8.5)	.18	
Vascular change	1 (1.5)	3 (5.1)	.25	
Findings consistent with chronic inflammation	25 (37.9)	27 (45.8)	.37	
Chronic villitis	8 (12.1)	3 (5.1)	.16	
Chronic chorioamnionitis	3 (4.5)	0	.09	
Chronic deciduitis	25 (37.9)	26 (44.1)	.48	

Continuous variables are presented as mean ± SD. Categorical variables are presented as number (proportions). *aP* value-adjusted by gestational age at delivery

We analyzed the perinatal outcomes for PP according to presence of APH (Table 5). In maternal pregnancy outcomes, the PP with APH group delivered earlier (36.0 ± 2.6 vs. 37.5 ± 1.3 weeks, *P* < 0.0001), had higher rate of emergency cesarean section, postpartum hemorrhage, postpartum anemia, and massive transfusion (63.6% vs. 10.2%, *P* < 0.0001; 60.6% vs. 32.2%, *P* = 0.002; 45.5% vs. 18.6%, *P* = 0.002; and 27.3% vs. 8.5%, *P* = 0.01, respectively). Furthermore, a higher composite adverse pregnancy outcome was observed in the PP with APH group (83.3% vs. 49.2%, *P* = 0.0001). When neonatal outcomes were compared between the two groups, the neonates from the PP with APH group had a significantly higher rate of neonatal complications. The mean neonatal birth weights were 2615.8 ± 538.5 g in the PP with APH group and 3010.6 ± 468.9 g in the PP without APH group. Among 66 neonates from the APH group, neonatal death occurred in one case due to early preterm delivery; the gestational age at delivery was 24 + 2 weeks. Preterm birth, NICU admission, use of mechanical ventilation, and sepsis were more common in the neonates of the PP with APH group (49.9% vs. 11.9%, *P* = 0.0001; 56.1% vs. 22.0%, *P* = 0.0001; 25.8% vs. 11.9%, *P* = 0.04; and 31.8% vs. 13.6%, *P* = 0.01, respectively). Moreover, a higher composite adverse neonatal outcome was observed in the PP with APH group (59.1% vs. 23.9%, *P* = 0.0001).

**Table 5:** Perinatal outcomes

	Placenta previa with APH (*N* = 66) *n*/*N* (%)	Placenta previa without APH (*N* = 59) *n*/*N* (%)	*P* value	*aP* value
Maternal pregnancy outcomes				
Maternal death	0	0	N/A	
Gestational age at delivery (weeks)	36.0 ± 2.6	37.5 ± 1.3	< .0001	
Emergency cesarean section	42 (63.6)	6 (10.2)	< .0001	
Placenta abruption	1 (1.5)	0	.34	
Postpartum hemorrhage	40 (60.6)	19 (32.2)	.002	
Cesarean hysterectomy	3 (4.5)	1 (1.7)	.36	
Postpartum anemia (Hb < 8)	30 (45.5)	11 (18.6)	.002	
Massive transfusion	18 (27.3)	5 (8.5)	.01	
Postoperative ICU admission	4 (6.1)	3 (5.1)	.81	
Composite adverse pregnancy outcome^†^	55 (83.3)	29 (49.2)	.0001	
Neonatal outcomes				
Neonatal death	1 (1.5)	0	.34	N/A
Preterm birth	29 (43.9)	7 (11.9)	.0001	N/A
Birth weight (grams)	2615.8 ± 538.5	3010.6 ± 468.9	< .0001	.002
Apgar 5 min < 7	2 (3.0)	0	.17	N/A
SGA	10 (15.2)	6 (10.2)	.41	.11
NICU admission	37 (56.1)	13 (22.0)	.0001	.003
Mechanical ventilation apply	17 (25.8)	7 (11.9)	.04	.38
Respiratory distress syndrome	14 (21.2)	6 (10.2)	.09	.66
Intraventricular hemorrhage	1 (1.5)	0	.34	N/A
Necrotizing enterocolitis	2 (3.0)	1 (1.7)	.62	.81
Sepsis	21 (31.8)	8 (13.6)	.01	.03
Seizure	1 (1.5)	0	.34	N/A
Composite adverse neonatal outcome^‡^	39 (59.1)	14 (23.9)	.0001	.007

Continuous variables are presented as mean ± SD. Categorical variables are presented as number (proportions). *aP* value-adjusted by gestational age at delivery

*Abbreviation*s: *SGA* small for gestational age, *NICU* neonatal intensive care unit, *N/A* not applicable

^†^Composite adverse pregnancy outcomes included any of the following: maternal death, emergency caesarean section, placenta abruption, postpartum hemorrhage, intrauterine tamponade use, cesarean hysterectomy, postpartum anemia (Hb < 8), massive transfusion, and postoperative ICU admission

^‡^Composite adverse neonatal outcomes included any of the following: neonatal death, preterm birth, Apgar scores at 5 min < 7, SGA, NICU admission, mechanical ventilation, respiratory distress syndrome, intraventricular hemorrhage, necrotizing enterocolitis, sepsis, and seizure

## Discussion

PP is an important pregnancy-related disorder because it causes APH, abnormally invasive placenta, severe intrapartum and postpartum hemorrhage, preterm delivery, and perinatal mortality and morbidity [2, 6]. Among these, APH is the most critical complication because it is highly associated with adverse maternal and neonatal outcomes.

The primary goal of this study was to evaluate the risk factors associated PP with APH. To this end, we analyzed the clinical factors and ultrasonographic findings of PP in the presence or absence of APH. We discovered that the PP with APH group had a higher incidence of antepartum uterine contractions, leading to greater use of tocolytics and steroids. Yinka et al. reported that uterine contractions and cervical effacement cause separation of the placenta, which leads to APH [1]. This bleeding may stimulate further uterine contractions; repetition of this process may lead to massive bleeding or worse pregnancy outcomes.

Several studies have reported on ultrasound findings related to PP; however, studies on the ultrasound findings associated with APH in PP are rare. We divided the antepartum ultrasonographic findings of PP into placenta type, placentation site, adherent placenta, and cervical length and performed systematic analysis. The ultrasonographic findings showed that the type of PP, placentation site, and adherent placenta were not significantly associated with APH. A short cervical length at admission (< 2.5 cm) was, however, strongly associated with APH. Previous studies have reported that a short cervix is a risk factor in predicting massive bleeding for PP [17–20]. Although the cut-off value was different in each study, we used 2.5 cm as the standard length for a short cervix. Therefore, to predict APH in PP, it is essential to follow-up the cervical length along with uterine contractions.

The second goal of the current study is to reveal the placental pathologic change and perinatal outcomes. We compared placental pathology between the two groups. The placental gross findings revealed that PP with APH group has lower placental weight, smaller chorionic diameters, and reduced chorionic plate area than the PP without APH group. The PP with APH group had higher rates of preterm birth. The same results were observed when we re-analyzed the data with adjustment for the gestational age. In the histopathological findings, the PP with APH group was associated with maternal malperfusion, including villous agglutination. Weiner et al. studied placental pathology and pregnancy outcome in PP, and reported similar findings as our results, that PP with APH was associated with a smaller placenta and a higher rate of villous changes related to maternal malperfusion lesions [21, 22].

Many studies have shown that APH is related to adverse maternal and neonatal outcomes consisting of massive intrapartum bleeding, PPH, hysterectomy, preterm delivery, higher rates of NICU admission, low birth weight, and other adverse neonatal outcomes [21–24]. Our results showed that the PP with APH group had a higher rate of emergency cesarean delivery, PPH, postpartum anemia, and blood transfusion. Abnormal placentation and placenta malperfusion due to bleeding might induce placental pathologic changes and affect fetal growth and neonatal outcomes. Regarding the neonatal outcomes, the frequency of neonatal complications such as preterm birth, low birth weight, and NICU admission was higher in the PP with APH group. There were also significant differences between the two groups when the analysis was performed after adjusting for gestational age at delivery.

Our study has several strengths. To the best of our knowledge, there are no comprehensively analyzed studies on clinical factors and ultrasound findings for complications of PP, especially for APH. We therefore performed an integrated analysis of risk factors related to APH in PP and resultant placental change and perinatal outcomes. We ensured the reliability of this study’s results by consistently maintaining patient data management, diagnosis and reporting clinicians, and the ultrasound team. In addition, a placental pathology examination was performed by two perinatal pathologist experts. However, this study has some limitations. It was a single-center study and the data collection period was short (3 years), resulting in a small sample size. Future multicenter studies with longer periods of data collection data could compensate for the above limitation.

## Conclusion

In conclusion, PP with APH was associated with more complications, a higher rate of preterm birth, increased postpartum hemorrhage, and worse neonatal outcomes than PP without an episode of APH. Although APH could not be predicted in women with PP, preterm uterine contraction and short cervical length were the most significant risk factors for PP with APH. Considering these factors, we believe that it is necessary to manage patients with PP with a higher level of caution.

## Data Availability

The data that support the findings of this study are available on request from the corresponding author. The data are not publicly available due to privacy or ethical restrictions.
